# Role of Statins after Endovascular Repair of Abdominal Aortic Aneurysms: A Nationwide Population-Based Study

**DOI:** 10.3390/jcm12113737

**Published:** 2023-05-29

**Authors:** Gyu Chul Oh, Kwan Yong Lee, Eun Ho Choo, Byung-Hee Hwang, Wook Sung Chung, So-Jeong You, JinKyung Jeon, Sol Kwon, Kiyuk Chang

**Affiliations:** 1 Department of Internal Medicine, Seoul St. Mary’s Hospital, The Catholic University of Korea, Seoul 06591, Republic of Korea; david.gyuchul@gmail.com (G.C.O.); cycle210@catholic.ac.kr (K.Y.L.);; 2 Catholic Research Institute for Intractable Cardiovascular Disease (CRID), College of Medicine, The Catholic University of Korea, Seoul 06591, Republic of Korea; 3 Medtronic Korea, Ltd., Seoul 06181, Republic of Koreajinkyung.jeon@medtronic.com (J.J.);

**Keywords:** abdominal aortic aneurysm, endovascular aneurysm repair, statin, mortality

## Abstract

The role of statins after endovascular abdominal aortic aneurysm repair (EVAR) has not been well established in an Asian context. In this study, the use of statins and their association with long-term health outcomes were evaluated in patients undergoing EVAR using the Korean National Health Insurance Service database. Among the 8893 patients who underwent EVAR from 2008 to 2018, 3386 (38.1%) were on statins prior to the procedure. Patients using statins had a higher prevalence of comorbidities, such as hypertension (88.4% vs. 71.5%), diabetes mellitus (24.5% vs. 14.1%), and heart failure (21.6% vs. 13.1%), compared with non-users (all *p <* 0.001). After propensity score matching, statin use prior to EVAR was associated with a lower risk of all-cause mortality (HR 0.85, 95% CI 0.78–0.92, *p <* 0.001) and cardiovascular mortality (HR 0.66, 95% CI 0.51–0.86, *p =* 0.002). Statin use following EVAR was associated with a lower risk of adverse events, but not significantly so. Patients on statins both preceding and following EVAR had a lower risk of all-cause mortality (HR 0.82, 95% CI 0.73–0.91, *p <* 0.001) and cardiovascular mortality (HR 0.62, 95% CI 0.44–0.87, *p =* 0.007), compared with statin non-users. Among Korean patients undergoing EVAR, the persistent use of statins prior to and after the procedure was associated with a lower risk of mortality, compared with non-statin users.

## 1. Introduction

Treatment for abdominal aortic aneurysm (AAA) has historically been with open surgical repair. Since the first reports of endovascular repair using a stent graft by Volodos [1] and Parodi [2], innovations in equipment and procedure technology have enabled physicians to perform endovascular repairs, achieving results comparable to surgical repairs. In patients undergoing endovascular repair, factors such as old age, sex (female), and comorbidities have been shown to influence outcomes [3,4,5].

In addition to EVAR, medical treatment plays a significant role in the management of AAA. Among the various pharmacological agents used, statins have gained considerable attention due to their pleiotropic effects beyond their lipid-lowering properties [6]. In recent years, statin use has proven its efficacy in reducing mortality in high-risk patients [7]. Due to its benefit in reducing cardiovascular events, guidelines recommend intensive statin therapy to lower low-density-lipoprotein cholesterol (LDL-C) in patients with established atherosclerotic cardiovascular disease (ASCVD) [8,9]. Currently, statins have demonstrated favorable outcomes in cases of atherosclerotic disease and have been widely used for primary and secondary prevention measures. However, the role of statins in the management of patients with abdominal aortic aneurysms has not been widely evaluated. Statin use after endovascular repair surgery has been reported to help stabilize the aortic sacs and to be associated with the regression of type 2 endoleaks [10]. A meta-analysis of 11,933 patients has also shown that statins have a moderate effect in reducing mortality after aneurysm repair, but this result was mostly derived from studies considering both endovascular and surgical repair [11].

Although several trials have reported favorable outcomes associated with statin use after AAA repair, most of the studies were performed in a Western population. Due to the increasing prevalence of AAA in Asian populations, there has been a need for a dedicated study of an Asian AAA cohort. Therefore, this study aims to evaluate the association between statin use and cardiovascular outcomes following EVAR in Asian patients with AAA. The current study was designed to evaluate data from a nationwide health insurance database and assess the relationship between statin use and long-term outcomes in patients who are exclusively undergoing endovascular AAA repair (EVAR). Statin use was investigated at both the pre- and post-treatment periods and its association with outcomes was evaluated. By analyzing a large nationwide population-based dataset, we aim to assess the optimal role of statins in post-EVAR treatment, providing valuable insights for clinical decision-making and the potential improvement of patient outcomes.

## 2. Materials and Methods

### 2.1. Study Design

This was a retrospective, population-based cohort study using the Korean National Health Insurance Service (NHIS) database. The Korean NHIS is a government-run, mandatory insurance service that covers the whole population. The NHIS records patient demographic data and claims data for medical services, including surgeries, procedures, and prescription medications. Information is provided anonymously to investigators for research purposes after approval of the study protocol by the institution’s review committee.

The study protocol was reviewed and approved by the Institutional Review Board of the Catholic University of Korea, Seoul St. Mary’s Hospital (KC20ZISI0537), and written consent was waived due to the retrospective design of the study. The study was performed in accordance with the Declaration of Helsinki.

### 2.2. Study Population

**Figure 1:**
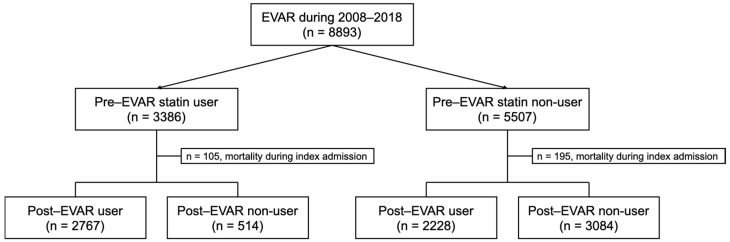
The study design. Note: EVAR, endovascular aneurysm repair.

Patients over 18 years of age who were undergoing endovascular repair for unruptured AAA were screened. Diagnoses were classified using the International Classification of Diseases, 10th Revision, Clinical Modification (ICD-10-CM) ICD-10 codes. Diagnosis of unruptured AAA was defined according to the ICD-10 codes I71.4, I71.6, and I71.9. Treatment by endovascular repair was defined as procedure codes M6611, M6612, or M6613 during an admission period. Pre-treatment statin use was defined as having at least 2 separate prescriptions of any statins 6 months prior to the index admission. Post-treatment statin use was defined as statin prescription at discharge, plus at least 1 prescription of statins at an outpatient visit during the 1 month after discharge. The design of the study is shown in Figure 1.

### 2.3. Definition of Variables and Outcomes

The primary outcome was defined as all-cause mortality. Secondary outcomes were cardiovascular mortality, reintervention or the surgical treatment of aneurysms, myocardial infarction, systemic embolism, and stroke. Definitions of outcomes used in the study are provided in Table S1 in the Supplementary Materials.

The perioperative risk of patients undergoing EVAR was calculated using the Medicare perioperative risk score (MPRS) [12]. Patients with an MPRS of <3 were defined as low-risk patients, those with scores from 3 to 11 were defined as intermediate-risk patients, and individuals with scores of ≥11 were defined as high-risk patients.

### 2.4. Statistical Analysis

Continuous variables are expressed as mean ± standard deviation or median (interquartile range) and categorical variables are described in numbers and percentages. To control the confounding factors between the use of statins and outcomes, we performed 1:1 propensity-score (PS)-matching between pre-EVAR statin user and non-user groups. The score was calculated by a multiple logistic regression model including age, sex, hypertension, diabetes, heart failure, ischemic heart disease, atrial fibrillation, chronic obstructive pulmonary disease, peripheral artery disease, chronic kidney disease, stroke, and preoperative risk score as independent variables. The balance of covariates between the groups was evaluated using the standard mean difference (SMD); the SMDs of all covariates except the baseline myocardial infarction were less than 0.1. To evaluate the association between pre-EVAR statin use and outcomes, the Cox proportional hazards regression model, along with a robust variance estimator, was used for the matched data and included baseline myocardial infarction as an adjustment variable. Characteristics among the four groups according to pre- and post-EVAR statin use were compared, using an analysis of variance (ANOVA) for continuous variables or the chi-square test for categorical variables. Cox proportional hazards regression analysis was used to assess the outcomes between groups, incorporating age, sex, hypertension, diabetes, heart failure, ischemic heart disease, atrial fibrillation, chronic obstructive pulmonary disease, peripheral artery disease, chronic kidney disease, and stroke as covariables. All statistical analyses were performed using the SAS Enterprise Guide 7.15 (SAS Institute Inc., Cary, NC, USA) or R version 4.1.2 (R Foundation for Statistical Computing, Vienna, Austria), and 2-sided *p*-values of < 0.05 were considered significant.

## 3. Results

### 3.1. Patient Characteristics

Among the 8893 patients undergoing EVAR from 2008 to 2018, 3386 (38.1%) were using statins prior to the procedure. The mean age of patients was 73.0 ± 8.4 years and the median follow-up was 38.7 months (interquartile range (IQR) 19.9–66.2 months). Patients prescribed statins before undergoing EVAR had a higher prevalence of comorbidities such as hypertension (88.4% vs. 71.5%), diabetes mellitus (24.5% vs. 14.1%), and heart failure (21.6% vs. 13.1%), compared with non-users (all *p* < 0.001). The perioperative risk calculated by MPRS was also significantly higher in pre-treatment statin users. The baseline characteristics of the study cohort according to statin use are described in Table S2 in the Supplementary Materials.

To adjust for significant differences in baseline characteristics, 1:1 PS matching was performed, according to statin use. After PS matching, there were no significant differences in characteristics between groups except for the history of myocardial infarctions in patients with pre-EVAR statin use. Among a total of 6242 matched patients with a median follow-up period of 37.2 months (IQR 19.5–63.8 months), the mean age was 72.9 ± 8.2 years, and 82.6% of the patients were men. A high proportion of patients were on antihypertensive medications (88.2%), while 51.5% of patients had a history of ischemic heart disease. The median MPRS was 6.0 (IQR 1–11), with 46.6% having intermediate or high perioperative risk. The patient characteristics of the matched population are described in Table 1.

### 3.2. Outcomes According to Pre-Treatment Statin Use

In the PS-matched population, patients on statins prior to EVAR were associated with a significantly lower incidence of all-cause mortality (incidence rate 83.9 per person-years (PPY) vs. 98.9 PPY, HR 0.85, 95% CI 0.78–0.92, *p* < 0.001) and cardiovascular mortality (HR 0.66, 95% CI 0.51–0.86, *p* = 0.002) compared with non-users (see Figure S1 in the Supplementary Materials*)*. For short-term outcomes, the 30-day mortality rate was not significantly different between groups (HR 1.15, 95% CI 0.84–1.58, *p* = 0.376) (see Figure S2 in the Supplementary Materials). There was no significant difference either in reintervention rates, incidence of myocardial infarction, or stroke between groups. The significant difference in all-cause and cardiovascular mortality were also observed in the non-matched population. The results of the Cox proportional hazards regression analyses for the PS-matched and the whole population are shown in Table 2 and Table S3 in the Supplementary Materials.

### 3.3. Outcomes According to Post-Operative Statin Use

Among the 8593 patients surviving until discharge after EVAR, 58.1% (4995 of 8593) were defined as post-treatment users of statins, according to the prespecified criteria. After PS-matching, statin users following EVAR showed a numerically lower number in terms of all-cause mortality (HR 0.97, 95% CI 0.89–1.06, *p* = 0.494) and cardiovascular mortality (HR 0.85, 95% CI 0.64–1.13, *p* = 0.252), but not significantly so.

**Figure 2:**
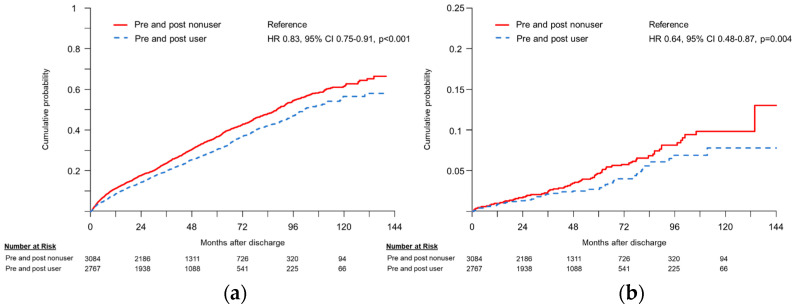
Kaplan-Meier curve for (**a**) all-cause mortality and (**b**) cardiovascular mortality according to pre- and post-EVAR statin use.

When patients were grouped according to pre- and post-treatment statin use, those using statins both preceding and following EVAR had a higher prevalence of comorbidities, such as hypertension and ischemic heart disease, compared with other groups (Table 3). However, these patients showed significantly lower rates of all-cause mortality (HR 0.82, 95% CI 0.73–0.91, *p* < 0.001) and cardiovascular mortality (HR 0.62, 95% CI 0.44–0.87, *p* = 0.007) compared with those without any statin use (Figure 2a). Furthermore, patients with continuous statin use had a lower rate of cardiovascular mortality compared with those discontinuing statins after the procedure (Figure 2b). No differences were observed for the incidence of reintervention, myocardial infarction, or stroke according to peri-EVAR statin use. The results of Cox regression analyses are provided in Table S4 in the Supplementary Materials.

### 3.4. Subgroup Analysis

Mortality outcomes were compared according to the different patient subgroups and indicated that the incidence of all-cause mortality and cardiovascular mortality did not differ according to sex, age, the presence of hypertension, diabetes, or preoperative risk in patients undergoing endovascular repair. The results of the subgroup analyses for all-cause mortality and cardiovascular mortality are shown in Figure S3 in the Supplementary Materials.

## 4. Discussion

Using a large, nationwide health insurance claims database, we analyzed the data from 8893 Korean patients undergoing endovascular repair of an unruptured AAA and showed that maintaining statin use both pre- and post-procedure was associated with lower rates of mortality. After multivariate Cox regression analyses and PS-matching, patients using statins before the EVAR procedure showed lower rates of all-cause and cardiovascular mortality, although they exhibited higher preoperative risk. The novelty of this study is that we assessed patient outcomes according to pre- and post-EVAR statin use and showed that persistent use before and after the EVAR procedure was associated with more favorable mortality outcomes. However, statin use was not associated with differences in terms of the risk of reintervention, myocardial infarction, or stroke.

The role of statins has been widely investigated in patients with coronary heart disease. Lowering LDL-C with statins has been confirmed to reduce cardiovascular mortality and morbidity in patients with coronary artery disease or in those at high risk of developing atherosclerotic cardiovascular disease [13]. Statin use in the perioperative period has also been shown to be associated with favorable outcomes. In a retrospective analysis of 180,478 veterans undergoing noncardiac surgeries, early exposure to statins was associated with a significant reduction in all-cause mortality and cardiovascular complications [14]. Although current guidelines recommend the prescribing of statins to reduce comorbid cardiovascular diseases for patients with AAA, there is as yet no concrete evidence supporting this recommendation. Furthermore, the emphasis in medical treatment lies in preventing AAA expansion; however, the data are lacking on the management of patients after aneurysm repair [15].

Using the EUROSTAR registry, Leurs et al. employed multivariate regression models (81% vs. 77%, *p* = 0.005) [16] to reveal that pre-EVAR statin users were associated with a higher survival rate compared with non-users. Conversely, analysis of the registry data of 3217 patients showed that hypercholesterolemia was not a significant predictor of early mortality in patients undergoing AAA repair. A meta-analysis including 134,290 patients showed that statin use was associated with a significant decrease in both long-term and short-term mortality after aneurysm repair [17]. However, the included studies were mostly performed in the United States or Europe, not fully representing the Asian population. In our cohort of Korean patients undergoing EVAR, pre-procedure statin use was associated with a lower rate of total mortality and cardiovascular mortality, but no significant difference was observed in terms of 30-day mortality.

Although statins are effective in preventing future cardiovascular events, the persistence rates are quite low, even in high-risk patients. In patients with diabetes or atherosclerotic cardiovascular disease, five-year statin persistence was less than 25% [18]. Among 158,795 patients hospitalized for myocardial infarctions, less than 50% of patients showed a high persistence rate (≥80% use) in the following 6 months after discharge [19]. We hypothesized that patients discontinuing statins after AAA repair would have a different prognosis, compared with those maintaining statin use, and investigated whether statins were maintained after the procedure. Patients taking statins both before and after EVAR had a higher preoperative risk and an increased prevalence of comorbidities, even when those not surviving until discharge were excluded from the analysis. Even so, statin persistence was associated with a significantly lower rate of mortality after adjusting for comorbidities.

 While several studies have shown that long-term mortality significantly decreased after aneurysm repair in statin users, a subgroup analysis of a previous meta-analysis has reported that no difference in mortality was observed in studies with an exclusively Asian population [17]. However, the studies that were included had only a limited number of patients and were single-center retrospective analyses [20,21]. We were able to recruit a large retrospective cohort of patients undergoing EVAR, due to the Korean NHIS database. The Korean NHIS is a mandatory, government-run system, and the records of all patient prescriptions and procedures are registered. The results of the current analysis showed that statin use was associated with a lower risk of all-cause and cardiovascular mortality in Korean patients also. Further pooled analyses may be needed to assess the possibility of an ethnic difference associated with statin use.

 Similar to the current study, Liao et al. reported outcomes in the context of statins using a nationwide database from Taiwan. Post-treatment statin use, which was defined as persistent statin use 30 days after the procedure, was associated with a significantly lower mortality rate. However, only 12.2% (199/1633) of the whole study population were prescribed statins after AAA repair [22]. In our cohort, 38.1% (3386/8893) were using statins before EVAR and, among them, 81.7% maintained statin use after the procedure. Furthermore, more than 60% of the patients were categorized as being at intermediate to high risk, according to the MPRS. The role of statins may have been more pronounced in our cohort of patients at high cardiovascular risk. A plausible explanation for the observed survival benefit in individuals who continued statin use after EVAR may relate to their higher prevalence of indications for statin therapy, as evidenced in the patient characteristics based on pre- and post-EVAR statin use. Patients who maintained statin use displayed a numerical increase, albeit not one that was statistically significant, in the prevalence of prior myocardial infarctions, diabetes, and hypertension. While these comorbidities themselves are associated with elevated risks, the sustained use of statins may have ameliorated their detrimental effects, ultimately yielding long-term benefits.

### Limitations

The limitations of the study are as follows. First, the current study analyzed the claims data from a national health insurance service. Although a large number of patients were recruited, data on the biochemistry and anatomical features of aneurysms were not available for analysis. As in patients with coronary artery disease, on-treatment LDL-C levels might have been a significant factor associated with the outcomes. Second, there are limitations in the retrospective design of the study. The causal relationship between statins and mortality in patients undergoing EVAR cannot be assessed with the current study, and prospective, randomized trials would be needed to better understand the relationship between statins and outcomes. Third, there was a significant difference in the baseline characteristics between statin users and non-users. There may have been a tendency for the increased use of statins according to comorbidities. As patients with AAA are also at higher risk of cardiovascular disease, statin use may have influenced these outcomes [23]. To adjust for the differences in outcomes, the investigators performed Cox regression analyses using covariates and performing PS-matching and demonstrated consistent results. Fourth, the outcomes were defined by diagnostic/treatment codes. Although previous studies have clearly defined and validated these outcomes, this might have caused an overestimation of event rates. Fifth, due to the definition used to assess post-EVAR statin use, cases of early fatalities were excluded from the analysis, which could have an impact on the outcomes. Sixth, the dose, intensity, and compliance of the statins used were not accounted for in the analysis. Although a retrospective analysis of 6497 patients undergoing open AAA repair reported that statin intensity was not associated with in-hospital morbidity or mortality [24], future studies will be needed to evaluate the optimal intensity or target LDL-C levels in patients undergoing EVAR.

## 5. Conclusions

The current study investigated the role of statin use among Korean patients undergoing EVAR for unruptured AAAs. The results demonstrated that pre-EVAR statin use was associated with a significantly lower incidence of all-cause mortality and cardiovascular mortality. However, short-term outcomes and the incidences of myocardial infarction and stroke did not differ significantly between statin users and non-users. Furthermore, continuous statin use, both before and after EVAR, was associated with lower rates of cardiovascular mortality compared to that seen in those discontinuing statins after the procedure. These findings suggest that statin therapy may have a beneficial effect in reducing mortality in patients undergoing EVAR for AAA.

Our study contributes to the growing body of evidence on the potential benefits of statin use in the management of AAA. These results support the notion that statins, which are commonly used for the primary and secondary prevention of atherosclerotic disease, may have additional benefits in the context of EVAR. Further research is warranted to explore the underlying mechanisms and optimal strategies for statin use in this patient population.

## Figures and Tables

**Table 1:** Baseline characteristics according to pre-EVAR statin use in the PS-matched population.

	Pre-EVAR Statin User (*n* = 3121)	Pre-EVAR Statin Non-User (*n* = 3121)	SMD	*p*-Value ^1^
Age (years ± SD)	72.9 ± 7.7	73.0 ± 8.7	0.003	0.905
Sex (male, *n* (%))	2585 (82.8)	2573 (82.4)	0.010	0.689
MPRS	6.5 ± 6.0	6.5 ± 6.0	0.027	0.993
Low risk	1075 (34.4)	1062 (34.0)		
Intermediate risk	1432 (45.9)	1475 (47.3)		
High risk	614 (19.7)	584 (18.7)		
Comorbidities				
Hypertension	2729 (87.4)	2753 (88.2)	0.024	0.353
Diabetes mellitus	675 (21.6)	645 (20.7)	0.024	0.304
Heart failure	614 (19.7)	582 (18.6)	0.026	0.304
Ischemic heart disease	1616 (51.8)	1599 (51.2)	0.011	0.667
Myocardial infarction	423 (13.6)	259 (8.3)	0.169	<0.001
Atrial fibrillation	253 (8.1)	247 (7.9)	0.007	0.780
COPD	391 (12.5)	375 (12.0)	0.016	0.537
Peripheral artery disease	454 (14.5)	472 (15.1)	0.016	0.522
Chronic kidney disease	77 (2.5)	80 (2.6)	0.006	0.808
Cerebrovascular disease	845 (27.1)	852 (27.3)	0.005	0.842

EVAR, endovascular aneurysm repair; PS, propensity score; SMD, standardized mean difference; SD, standard deviation; MPRS, Medicare perioperative risk score; COPD, chronic obstructive pulmonary disease. ^1^ *p*-value according to the conditional logistic regression model.

**Table 2:** Risks of outcomes according to pre-EVAR statin use in the PS-matched population.

	Pre-EVAR Statin User (*n* = 3121)		Pre-EVAR Statin Non-User (*n* = 3121)		HR	95% CI	*p*-Value
	No. of Events	Incidence Rate ^1^	No. of Events	Incidence Rate ^1^			
30-day mortality	82	2.7	71	2.3	1.15	(0.84–1.58)	0.376
Overall mortality	985	83.9	1104	98.9	0.85	(0.78–0.92)	<0.001
Cardiovascular mortality	95	8.1	132	11.8	0.66	(0.51–0.86)	0.002
Reintervention	223	19.8	238	22.3	0.89	(0.74–1.06)	0.189
Myocardial infarction	61	5.3	62	5.6	0.85	(0.60–1.20)	0.362
Major bleeding	99	8.6	99	9.0	0.95	(0.72–1.25)	0.697
Any stroke	434	39.8	421	40.4	1.01	(0.88–1.15)	0.915
Hemorrhagic stroke	105	9.1	123	11.1	0.83	(0.64–1.07)	0.147
Ischemic stroke	340	30.8	299	28.4	1.12	(0.95–1.30)	0.171

EVAR, endovascular aneurysm repair; PS, propensity score; HR, hazard ratio; CI, confidence interval. ^1^ Incidence rates are per 1000 person-years, except for 30-day mortality (per 100 person-month).

**Table 3:** Baseline characteristics according to pre- and post-EVAR statin use.

	Pre- and Post-User (*n* = 2767)	Pre-User But Post-Non-User (*n* = 514)	Pre-Non-User But Post-User (*n* = 2228)	Pre- and Post-Non-User (*n* = 3084)	*p*-Value ^1^
Age (years ± SD)	72.7 ± 7.7	72.7 ± 7.6	72.8 ± 8.3	73.0 ± 9.2	0.905
Sex (male, *n* (%))	2309 (83.4)	420 (81.7)	1896 (85.1)	2594 (84.1)	0.689
MPRS	6.6 ± 6.0	6.1 ± 5.8	5.8 ± 5.7	6.0 ± 5.8	0.993
Low risk	935 (33.8)	187 (36.4)	861 (38.6)	1176 (38.1)	
Intermediate risk	1283 (46.4)	239 (46.5)	1035 (46.5)	1427 (46.3)	
High risk	549 (19.8)	88 (17.1)	332 (14.9)	481 (15.6)	
Comorbidities					
Hypertension	2450 (88.5)	446 (86.8)	1643 (73.7)	2155 (69.9)	0.353
Diabetes mellitus	681 (24.6)	130 (25.3)	343 (15.4)	394 (12.8)	0.304
Heart failure	607 (21.9)	96 (18.7)	295 (13.2)	395 (12.8)	0.304
IHD	1556 (56.2)	259 (50.4)	893 (40.1)	900 (29.2)	0.667
MI	399 (14.4)	64 (12.5)	157 (7.0)	143 (4.6)	<0.001
Atrial fibrillation	236 (8.5)	45 (8.8)	133 (6.0)	209 (6.8)	0.780
COPD	341 (12.3)	66 (12.8)	271 (12.2)	423 (13.7)	0.537
PAD	414 (15.0)	68 (13.2)	316 (14.2)	427 (13.8)	0.522
CKD	69 (2.5)	8 (1.6)	37 (1.7)	63 (2.0)	0.808
CVD	761 (27.5)	173 (33.7)	458 (20.6)	623 (20.20)	0.842

EVAR, endovascular aneurysm repair; SD, standard deviation; MPRS, Medicare perioperative risk score; IHD, ischemic heart disease; MI, myocardial infarction; COPD, chronic obstructive pulmonary disease; PAD, peripheral artery disease; CKD, chronic kidney disease; CVD, cerebrovascular disease. ^1^ *p*-value by 2-way ANOVA.

## Data Availability

The data used in the current analysis can be obtained through the Korean National Health Insurance Service database. Investigators can submit study protocols and acquire the data for analysis after a board review.

## Supplementary Materials

The following supporting information can be downloaded at: https://www.mdpi.com/article/10.3390/jcm12113737/s1, Figure S1: Kaplan–Meier curve for (a) all-cause mortality, (b) cardiovascular mortality according to pre-EVAR statin use (PS-matched population); Figure S2: Kaplan–Meier curve for 30-day mortality according to pre-EVAR statin use; Figure S3: Subgroup analysis for all-cause mortality and cardiovascular mortality according to persistent statin use; Table S1: Definition of outcomes; Table S2: Baseline characteristics according to pre-EVAR statin use; Table S3: Risks of outcomes according to pre-EVAR statin use in the whole population; Table S4: Risk of outcomes according to pre- and post-EVAR statin use.
