# Supplementary material for: Role of Statins after Endovascular Repair of Abdominal Aortic Aneurysms: A Nationwide Population-Based Study
Source: J Clin Med. 2023 May 29;12(11):3737. doi: 10.3390/jcm12113737 (PMC10253659; doi:10.3390/jcm12113737)
Supplement: Supplementary file 1 [file jcm-12-03737-s001.zip › jcm-2362870-supplementary.pdf]

# Role of Statins after Endovascular Repair of Abdominal Aortic Aneurysms: A Nationwide Population-Based Study

**Gyu Chul Oh [1,2], Kwan Yong Lee [1,2], Eun Ho Choo [1,2], Byung-Hee Hwang [1,2], Wook Sung Chung [1,2], So-Jeong You [3], JinKyung Jeon [3], Sol Kwon [3] and Kiyuk Chang [1,2,*]**

[1] Department of Internal Medicine, Seoul St. Mary's Hospital, The Catholic University of Korea, Seoul 06591, Republic of Korea; david.gyuchul@gmail.com (G.C.O.); cycle210@catholic.ac.kr (K.Y.L.)
[2] Catholic Research Institute for Intractable Cardiovascular Disease (CRID), College of Medicine, The Catholic University of Korea, Seoul 06591, Republic of Korea
[3] Medtronic Korea, Ltd., Seoul 06181, Republic of Korea; jinkyung.jeon@medtronic.com (J.J.)
* Correspondence: kiyuk@catholic.ac.kr

## Supplementary Materials - Index

### Supplementary Figures and Tables

Supplementary Table S1 *pag. 2*
Supplementary Table S2 *pag. 3*
Supplementary Table S3 *pag. 4*
Supplementary Table S4 *pag. 5*
Supplementary Figure S1 *pag. 8*
Supplementary Figure S2 *pag. 9*
Supplementary Figure S3 *pag. 10*

**Supplementary Figures and Tables**

**Supplemental Table S1. Definition of outcomes**

| | **Definition** |
|---|---|
| Cardiovascular mortality | Death due to I00-99 |
| Reintervention | EVAR or OSAR after 3 months of index intervention |
| Myocardial infarction | Coronary stent placement and admission with I21, I22, or I25.2 |
| Systemic embolism | At least two different days of hospital visits (outpatient) or admission with I74 or N28.0 |
| Major bleeding | At least two different days of hospital visits (outpatient) or admission with I60-62, K25-28 (subcodes 0-2 and 4-6 only), K920-K922, K625, I850, I983 or D62 |
| Stroke | Brain CT, MRI, or MRA and admission with I60-64 (I60-62, hemorrhagic; I63, ischemic stroke) |

EVAR, endovascular aneurysm repair; OSAR, open surgical aneurysm repair; CT, computed tomography; MRI, magnetic resonance imaging; MRA, magnetic resonance angiography.

**Supplemental Table S2. Baseline characteristics according to pre-EVAR statin use**

| | Pre-EVAR statin user (n = 3,386) | Pre-EVAR statin nonuser (n = 5,507) | p-value* |
|---|---|---|---|
| Age (years ± SD) | 72.9 ± 7.7 | 73.0 ± 8.8 | 0.295 |
| Sex (male, n (%)) | 2,810 (83.0) | 4,631 (84.1) | 0.171 |
| MPRS | 6.6 ± 6.0 | 6.1 ± 5.8 | <0.001 |
| Low risk | 1,138 (33.6) | 2,071 (37.6) | |
| Intermediate risk | 1,569 (46.3) | 2,555 (46.4) | |
| High risk | 679 (20.1) | 881 (16.0) | |
| Comorbidities | | | |
| Hypertension | 2,994 (88.4) | 3,939 (71.5) | <0.001 |
| Diabetes mellitus | 830 (24.5) | 775 (14.1) | <0.001 |
| Heart failure | 732 (21.6) | 722 (13.1) | <0.001 |
| Ischemic heart disease | 1,873 (55.3) | 1,859 (33.8) | <0.001 |
| Myocardial infarction | 480 (14.2) | 314 (5.7) | <0.001 |
| Atrial fibrillation | 295 (8.7) | 365 (6.6) | <0.001 |
| COPD | 420 (12.4) | 724 (13.1) | 0.310 |
| Peripheral artery disease | 503 (14.9) | 770 (14.0) | 0.254 |
| Chronic kidney disease | 86 (2.5) | 116 (2.1) | 0.183 |
| Cerebrovascular disease | 971 (28.7) | 1,144 (20.8) | <0.001 |

EVAR, endovascular aneurysm repair; SD, standard deviation; MPRS, Medicare perioperative risk score; COPD, chronic obstructive pulmonary disease.
*p-value by conditional logistic regression model

**Supplemental Table S3. Risks of outcomes according to pre-EVAR statin use in the whole population**

| | pre-EVAR statin user (n = 3,386) | | pre-EVAR statin nonuser (n = 5,507) | | Adjusted HR† | 95% CI | p-value |
|---|---|---|---|---|---|---|---|
| | No. of events | Incidence rate* | No. of events | Incidence rate* | | | |
| 30-day mortality | 89 | 2.7 | 150 | 2.8 | 1.06 | (0.80 – 1.40) | 0.682 |
| Overall mortality | 1,067 | 84.4 | 2,115 | 100.7 | 0.83 | (0.77 – 0.90) | <.001 |
| Cardiovascular mortality | 101 | 8.0 | 219 | 10.4 | 0.65 | (0.51 – 0.83) | <0.001 |
| Reintervention | 240 | 19.8 | 428 | 21.3 | 0.91 | (0.77 – 1.07) | 0.258 |
| Myocardial infarction | 66 | 5.3 | 102 | 4.9 | 1.03 | (0.74 – 1.43) | 0.863 |
| Major bleeding | 111 | 8.9 | 169 | 8.1 | 1.07 | (0.83 – 1.38) | 0.610 |
| Any stroke | 480 | 41.0 | 739 | 37.7 | 0.93 | (0.88 – 1.05) | 0.235 |
| Hemorrhagic stroke | 114 | 9.1 | 210 | 10.1 | 0.85 | (0.67 – 1.08) | 0.192 |
| Ischemic stroke | 375 | 31.6 | 535 | 27.0 | 0.99 | (0.86 – 1.14) | 0.882 |

EVAR, endovascular aneurysm repair; HR, hazard ratio; CI, confidence interval.
*Incidence rates are per 1,000 person-years, except for 30-day mortality (per 100 person-month).
†Adjusted by age, sex, presence of hypertension, diabetes, heart failure, ischemic heart disease, atrial fibrillation, peripheral artery disease, chronic kidney disease, and cerebrovascular disease.

**Supplemental Table S4. Risk of outcomes according to pre- and post-EVAR statin use**

| | No. of subjects* | No. of events | Incidence rate (PPY) | Unadjusted HR (95% CI) | P† | Adjusted HR‡ (95% CI) | P† |
|---|---|---|---|---|---|---|---|
| *All-cause mortality* | | | | | | | |
| Pre and post nonuser | 3084 | 1178 | 95.9 | 1 (Ref.) | | 1 (Ref.) | |
| Pre nonuser but post user | 2228 | 742 | 87.5 | 0.92 (0.83-1.00) | 0.057 | 0.99 (0.90–1.09) | 0.846 |
| Pre user but post nonuser | 514 | 153 | 74.5 | 0.78 (0.66-0.92) | 0.004 | 0.79 (0.67–0.94) | 0.008 |
| Pre and post user | 2767 | 809 | 77.3 | 0.81 (0.74-0.88) | <.001 | 0.83 (0.76–0.91) | <.001 |
| *Cardiovascular death* | | | | | | | |
| Pre and post nonuser | 3084 | 122 | 9.9 | 1 (Ref.) | | 1 (Ref.) | |
| Pre nonuser but post user | 2228 | 75 | 8.8 | 0.90 (0.67-1.20) | 0.458 | 0.96 (0.72–1.28) | 0.778 |
| Pre user but post nonuser | 514 | 20 | 9.7 | 0.99 (0.62-1.59) | 0.960 | 0.85 (0.53–1.38) | 0.518 |
| Pre and post user | 2767 | 75 | 7.2 | 0.73 (0.55-0.97) | 0.030 | 0.64 (0.47–0.86) | 0.004 |
| *Reintervention* | | | | | | | |
| Pre and post nonuser | 3084 | 241 | 20.5 | 1 (Ref.) | | 1 (Ref.) | |
| Pre nonuser but post user | 2228 | 187 | 23.2 | 1.15 (0.95-1.39) | 0.148 | 1.15 (0.95–1.39) | 0.167 |

| | | | | | | | |
|---|---|---|---|---|---|---|---|
| Pre user but post nonuser | 514 | 35 | 17.8 | 0.88 (0.62-1.26) | 0.494 | 0.88 (0.61–1.26) | 0.475 |
| Pre and post user | 2766 | 204 | 20.4 | 1.02 (0.84-1.22) | 0.874 | 0.98 (0.80–1.19) | 0.811 |
| *Myocardial infarction* | | | | | | | |
| Pre and post nonuser | 3084 | 64 | 5.3 | 1 (Ref.) | | 1 (Ref.) | |
| Pre nonuser but post user | 2224 | 34 | 4.1 | 0.77 (0.50-1.16) | 0.206 | 0.78 (0.51–1.19) | 0.253 |
| Pre user but post nonuser | 514 | 16 | 7.9 | 1.49 (0.86-2.58) | 0.153 | 1.46 (0.84–2.55) | 0.181 |
| Pre and post user | 2767 | 49 | 4.7 | 0.89 (0.61-1.29) | 0.539 | 0.87 (0.59–1.28) | 0.469 |
| *Major bleeding* | | | | | | | |
| Pre and post nonuser | 3084 | 105 | 8.6 | 1 (Ref.) | | 1 (Ref.) | |
| Pre nonuser but post user | 2228 | 64 | 7.6 | 0.87 (0.64-1.19) | 0.389 | 0.94 (0.69–1.29) | 0.704 |
| Pre user but post nonuser | 514 | 15 | 7.4 | 0.85 (0.49-1.46) | 0.553 | 0.88 (0.51–1.52) | 0.656 |
| Pre and post user | 2767 | 96 | 9.3 | 1.07 (0.81-1.41) | 0.656 | 1.08 (0.80–1.44) | 0.622 |
| *Any stroke* | | | | | | | |
| Pre and post nonuser | 3025 | 364 | 31.8 | 1 (Ref.) | | 1 (Ref.) | |
| Pre nonuser but post user | 2178 | 255 | 32.1 | 1.00 (0.85-1.18) | 0.977 | 1.05 (0.89–1.23) | 0.573 |
| Pre user but post nonuser | 508 | 70 | 37.1 | 1.16 (0.90-1.50) | 0.253 | 1.02 (0.79–1.32) | 0.892 |

| | | | | | | | |
|---|---|---|---|---|---|---|---|
| Pre and post user | 2702 | 335 | 34.6 | 1.08 (0.93-1.25) | 0.323 | 0.98 (0.84–1.14) | 0.786 |
| *Hemorrhagic stroke* | | | | | | | |
| Pre and post nonuser | 3071 | 106 | 8.7 | 1 (Ref.) | | 1 (Ref.) | |
| Pre nonuser but post user | 2222 | 82 | 9.8 | 1.11 (0.83-1.48) | 0.479 | 1.15 (0.86–1.54) | 0.335 |
| Pre user but post nonuser | 514 | 18 | 8.9 | 1.01 (0.61-1.67) | 0.961 | 0.98 (0.59–1.62) | 0.934 |
| Pre and post user | 2758 | 87 | 8.4 | 0.95 (0.72-1.27) | 0.748 | 0.95 (0.71–1.28) | 0.753 |
| *Ischemic stroke* | | | | | | | |
| Pre and post nonuser | 3040 | 267 | 23.1 | 1 (Ref.) | | 1 (Ref.) | |
| Pre nonuser but post user | 2186 | 178 | 22.1 | 0.95 (0.79-1.15) | 0.607 | 1.01 (0.83–1.22) | 0.936 |
| Pre user but post nonuser | 508 | 53 | 27.5 | 1.19 (0.89-1.60) | 0.251 | 1.03 (0.77–1.39) | 0.838 |
| Pre and post user | 2713 | 258 | 26.3 | 1.13 (0.95-1.34) | 0.172 | 1.02 (0.85–1.22) | 0.852 |

PPY, per 1,000 person-years; HR, hazard ratio; Ref., reference; COPD, chronic obstructive pulmonary disease; PAD, peripheral artery disease; CKD, chronic kidney disease.

[†]p-value by Cox proportional hazards model

[‡] Adjusted by age, sex, presence of hypertension, diabetes, heart failure, ischemic heart disease, atrial fibrillation, peripheral artery disease, chronic kidney disease, and cerebrovascular disease.

**Supplemental Figure S1. Kaplan-Meier curve for (a) all-cause mortality, (b) cardiovascular mortality according to pre-EVAR statin use (PS-matched population)**

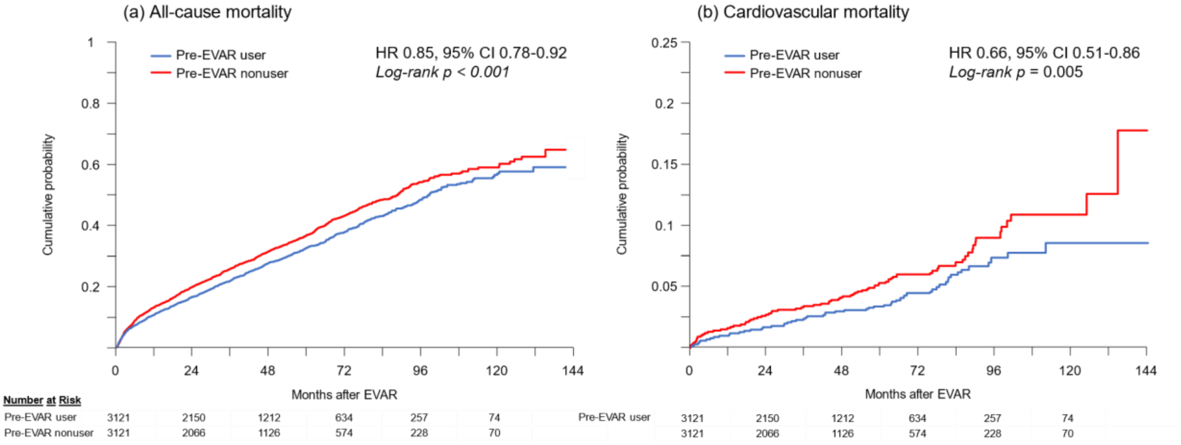


EVAR, endovascular repair; PS, propensity score.

**Supplemental Figure S2. Kaplan-Meier curve for 30-day mortality according to pre-EVAR statin use**

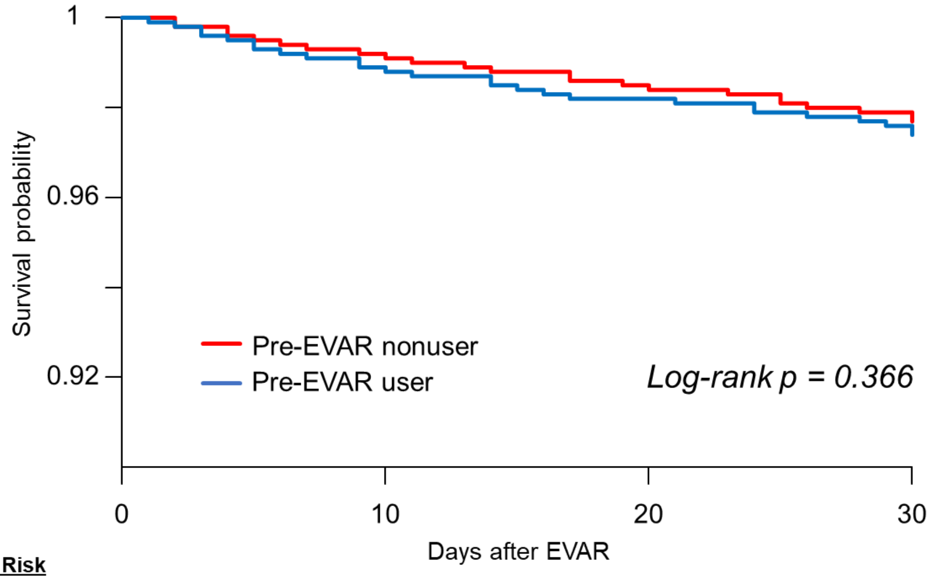


**Number at Risk**

| | | | | |
|---|---|---|---|---|
| Pre-EVAR user | 3121 | 3088 | 3064 | 3045 |
| Pre-EVAR nonuser | 3121 | 3096 | 3073 | 3054 |

EVAR, endovascular aneurysm repair

**Supplemental Figure S3. Subgroup analysis for all-cause mortality and cardiovascular mortality according to persistent statin use**

| Subgroup | Treatment | Placebo | All-cause mortality HR (95% CI) | All-cause mortality P-interaction* | CV mortality HR (95% CI) | CV mortality P-interaction* |
|---|---|---|---|---|---|---|
| Sex | | | | 0.800 | | 0.420 |
| Male | 2309 | 2594 | 0.84 (0.76 to 0.93) | | 0.74 (0.53 to 1.03) | |
| Female | 458 | 490 | 0.77 (0.62 to 0.96) | | 0.37 (0.19 to 0.75) | |
| Age | | | | 0.597 | | 0.733 |
| <80 years | 2219 | 2376 | 0.77 (0.69 to 0.86) | | 0.61 (0.43 to 0.88) | |
| ≥80 years | 548 | 708 | 0.86 (0.73 to 1.01) | | 0.62 (0.37 to 1.03) | |
| Preoperative risk | | | | 0.417 | | 0.434 |
| High | 549 | 481 | 0.94 (0.78 to 1.12) | | 0.64 (0.38 to 1.11) | |
| Medium | 1283 | 1427 | 0.81 (0.71 to 0.93) | | 0.55 (0.36 to 0.84) | |
| Low | 935 | 1176 | 0.73 (0.61 to 0.88) | | 0.92 (0.49 to 1.73) | |
| Hypertension | | | | 0.443 | | 0.628 |
| Yes | 2450 | 2155 | 0.86 (0.78 to 0.95) | | 0.66 (0.48 to 0.91) | |
| No | 317 | 929 | 0.67 (0.52 to 0.87) | | 0.68 (0.29 to 1.61) | |
| Diabetes mellitum | | | | 0.697 | | 0.746 |
| Yes | 681 | 394 | 0.86 (0.70 to 1.07) | | 0.66 (0.34 to 1.27) | |
| No | 2086 | 2690 | 0.82 (0.74 to 0.92) | | 0.65 (0.46 to 0.90) | |
| Ischemic heart disease | | | | 0.035 | | 0.628 |
| Yes | 1556 | 900 | 0.95 (0.83 to 1.09) | | 0.80 (0.53 to 1.21) | |
| No | 1211 | 2184 | 0.71 (0.62 to 0.82) | | 0.50 (0.31 to 0.80) | |
| Chronic kidney disease | | | | 0.704 | | 0.259 |
| Yes | 69 | 63 | 1.00 (0.65 to 1.55) | | 1.03 (0.11 to 10.18) | |
| No | 2698 | 3021 | 0.82 (0.75 to 0.91) | | 0.64 (0.47 to 0.87) | |
| Overall | 2767 | 3084 | 0.81 (0.74 to 0.88) | | 0.81 (0.74 to 0.88) | |

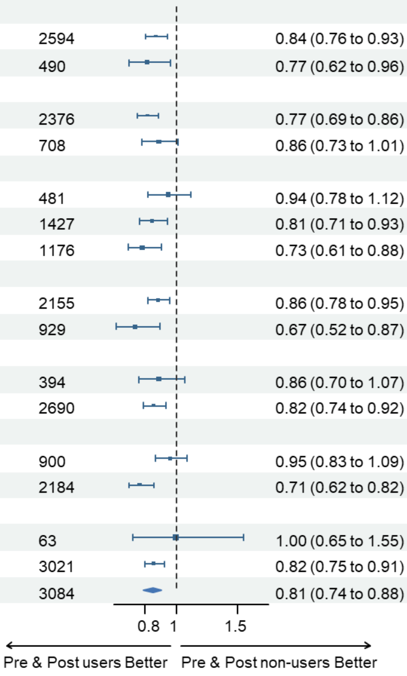


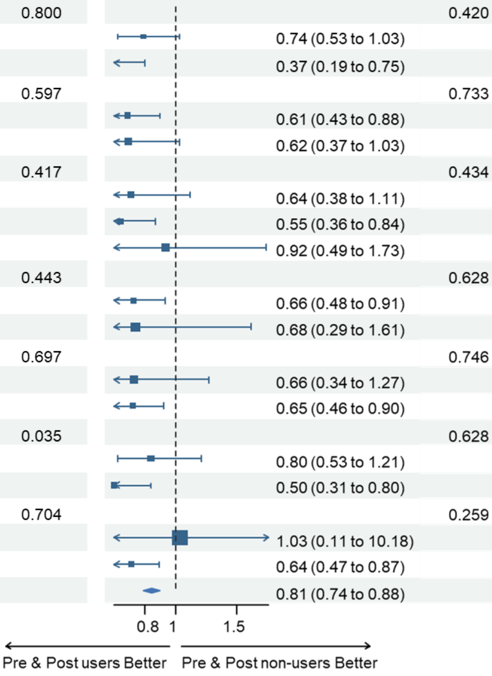


CV, cardiovascular; HR, hazard ratio.

*P-values were adjusted to account for multiple testing using the Benjamini-Hochberg method.
